# Disability from posttraumatic headache is compounded by coexisting posttraumatic stress disorder

**DOI:** 10.2147/JPR.S129808

**Published:** 2017-08-21

**Authors:** Louise S Roper, Peter Nightingale, Zhangjie Su, James L Mitchell, Antonio Belli, Alexandra J Sinclair

**Affiliations:** 1 Institute of Metabolism and Systems Research, College of Medical and Dental Sciences, University of Birmingham; 2 Centre for Endocrinology, Diabetes and Metabolism, Birmingham Health Partners; 3 Department of Neurology; 4 Wolfson Computer Laboratory, University Hospital Birmingham NHS Foundation Trust; 5 Institute of Inflammation and Ageing, College of Medical and Dental Sciences, University of Birmingham; 6 Health Research Surgical Reconstruction and Microbiology Research Centre, Birmingham, UK

**Keywords:** chronic headache, traumatic brain injury, neurotrauma

## Abstract

**Background:**

Posttraumatic headache (PTH) occurs in up to 82% of patients with traumatic brain injury (TBI). Posttraumatic stress disorder (PTSD) occurs in 39% of those with PTH. This study evaluates whether PTSD affects PTH disability.

**Methods:**

Eighty-six patients with TBI were prospectively evaluated in a secondary care trauma center. Headache disability was assessed using the Headache Impact Test version 6 and signs indicative of PTSD using the PTSD Check List Civilian version.

**Results:**

Increased PTSD-type symptoms were significantly associated with increased headache disability (*p*<0.001), as were employment status and loss of consciousness (*p*=0.049 and 0.016, respectively). Age was negatively correlated with headache disability (Spearman’s correlation rho=0.361, *p*=0.001).

**Conclusion:**

Increased severity of PTSD-type symptoms is significantly associated with increased headache disability in patients with chronic PTH. Managing PTSD symptoms in patients with chronic PTH may facilitate headache management.

## Introduction

PTH is defined as a headache with onset within 7 days of head trauma or within 7 days of regaining consciousness following head trauma.^1^ It has been reported to occur in 37%–82% of patients with TBI.^2^–^4^ Of these headaches, 47%–95% will persist for >3 months and are defined as chronic PTH.^5^ Chronic PTH is often persistent over at least 12 months,^2^,^6^ with >40% of patients with TBI reporting disability that persists at 1 year post-injury.^7^ Phenotypically, PTH resembles migraine in 49% of cases and tension-type headache in 40%, whereas 11% are undefined.^6^ Migraine is the seventh most disabling disease worldwide, and migraine following TBI is a major cause of disability.^8^ Subjects with new-onset PTH have decreased ability in memory tasks, slower reactions, and struggle in returning to their previous physical and social functions following TBI. Thus, PTH has a profound emotional, social, and economic impact.^3^,^9^,^10^

Headache disability following trauma may be worsened by the presence of comorbid PTSD and has already been documented as more pronounced in those with preexisting primary headaches.^9^–^11^ Pre-injury migraine may be the greatest predictor of PTH frequency and severity,^4^,^6^,^7^,^12^ with chronic migraineurs being at greater risk than those with episodic migraine.^13^ Gender, age, and severity of TBI have also been shown to influence PTH.^4^,^6^,^7^,^11^,^12^,^14^ Gender and severity of injury interact and have a complex effect on PTH. In the case of mild TBI, there is no difference in the incidence of PTH between men and women;^6^ however, PTH is reported to be more prevalent and persistent in women who have suffered moderate/severe injuries.^4^,^7^,^11^,^12^ Women may have greater headache-associated disability than men at 12 months,^4^,^7^ and differences in reporting of PTH between men and women persist at 11 years postinjury.^12^ Older patients have less incidence of PTH, but this may represent underreporting in this age group^6^ and hence requires further investigations.

Patients who have suffered TBI may have symptoms of PTSD. PTSD is a syndrome defined by the DSM-IV as a disorder related to actual or perceived harm to the individual or a close relative/friend or repeated exposure to aversive details of a traumatic event. Symptoms include intrusive memories, distressing dreams, flashbacks, psychological distress, and physiological adverse reactions.^15^ Reported prevalence of PTSD symptoms following TBI varies widely from 6% to 39% across both military and civilian populations.^3^,^12^,^16^ Headache may hinder recovery from the cognitive and behavioral consequences of TBI.^6^ Patients frequently have both PTH and PTSD symptoms,^3^,^6^,^12^,^16^ but the relationship between the symptoms is less defined.

In this study, we primarily hypothesize that headache disability following TBI is worsened by PTSD. Furthermore, we look at other factors that are associated with headache disability post-TBI.

## Methods

Consecutive patients with a history of TBI were prospectively seen in the TBI outpatient clinic at University Hospital Birmingham NHS Foundation Trust, UK, a secondary care trauma center, from December 2012 to July 2014. All patients with a TBI were eligible for this study. Patients were excluded if they failed to complete the headache or PTSD questionnaires. Ethical approval was given for the collection and use of these data by the Research and Audit Department of the University Hospital Birmingham, Queen Elizabeth. Verbal informed consent was obtained from all participants in this study. Data were collected over 19 months, but the data for each individual related to a single time point. Although informed consent was obtained from patients in order to collect these data and analyze them, express permission was not obtained to make the raw data public. The datasets are anonymous and can be provided as additional files if requested.

Headache disability was assessed using the Headache Impact Test version 6 (HIT-6).^17^ Scores were categorized into the following groups: severe impact (score ≥60), substantial impact (score 56–59), limited impact (score 50–55), and little to no impact (score ≤49).^17^ PTSD symptoms were assessed using PTSD Check List Civilian version (PCL-C).^18^,^19^ A score of >30 indicates the presence of PTSD symptoms.^20^ PCL-C is sanctioned by the National Institute for Health and Care Excellence for use in evaluating PTSD symptoms.^21^ It was chosen for use in this study due to its relatively few number of items and simple scoring system, which increases its reliability.^22^

Other factors investigated were the following: gender; educational level (high school or university); ethnicity; employment status (categories based on those used by the Office of National Statistics, UK^23^); interval from admission to hospital to filling in the questionnaire; whether an LOC occurred, either at the time of injury or subsequently up to and including the time of admission; whether seizures occurred, either associated with the injury or within 24 hours post-injury; GCS on admission (a score of <13 at admission defined a moderate/severe TBI);^1^,^24^ GOS;^24^ whether the patient was intoxicated with alcohol at the time of the injury; MOI (fall, road traffic accident, attack, home accident, self-harm, sports injury, or unknown mechanism); and severity of the injury on neuroimaging, quantified by the Marshall grading of patient’s first CT scan (scoring 1–6, with 1 being minimal injury and 6 being considerable).^25^

Statistical analyses were undertaken on IBM SPSS (version 20.0, released 2011). Data were not normally distributed, with the exception of age; therefore, nonparametric tests were employed in the main analysis. HIT-6 and PCL-C scores (noncategorical data) were assessed using Kruskal–Wallis and Mann–Whitney *U*-tests as appropriate. Spearman’s correlations were used to quantify the association between HIT-6 and PCL-C scores. HIT-6 and PCL-C categories (as described earlier) were used for graphical representation but not for statistical analysis. A Bonferroni correction was applied to account for multiple analyses. Data were further evaluated using a multiple linear regression model. Statistical significance was considered at *p*≤0.05.

## Results

**Figure 1:**
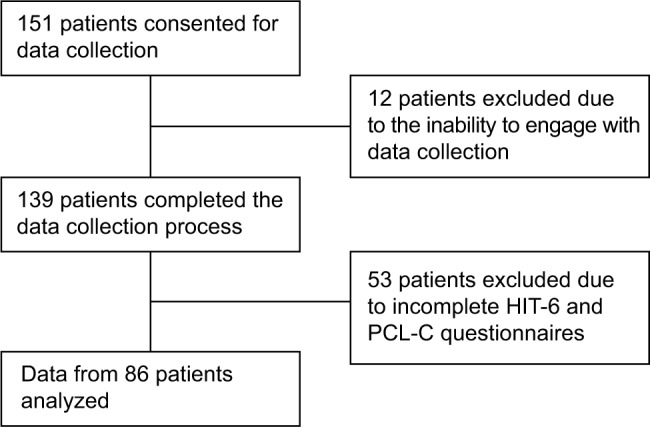
Diagram showing the selection of study patients. **Abbreviations:** HIT-6, Headache Impact Test version 6; PCL-C, PTSD Check List Civilian version; PTSD, posttraumatic stress disorder.

In total, 151 consecutive patients with TBI were approached in a TBI clinic. Twelve patients were not recruited due to the inability to engage with the study. The remaining 139 patients were recruited, but 53 were excluded because they did not complete the headache and PTSD questionnaires, which reflect the primary aims of this study. Therefore, data from 86 patients were analyzed (Figure 1). The majority of our cohort were white males (n=65, 75.6%) aged 45 ± 20 years. Patients in this study were evaluated at a median time of 5.6 months (170 days) post-injury in an outpatient clinic. Results are displayed in Table 1 and discussed further.

**Figure 2:**
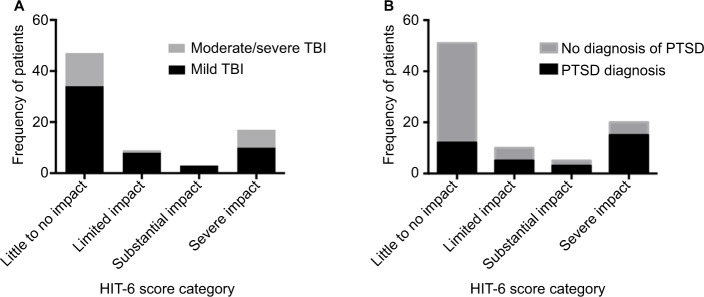
The number of patients with little, limited, substantial, and severe impact from PTH, as defined by their HIT-6 score. (**A**) Subcategorization by the number of patients with mild or moderate/severe TBI. (**B**) Subcategorization by the number of patients whose PCL-C scores indicate the presence/absence of PTSD symptoms. **Abbreviations:** PTH, posttraumatic headache; HIT-6, Headache Impact Test version 6; TBI, traumatic brain injury; PCL-C, PTSD Check List Civilian version; PTSD, posttraumatic stress disorder.

Fifty-five patients (64%) had a mild TBI, and 21 patients (24%) had suffered moderate/severe TBIs (data on severity of injury were not recorded in 10 participants). The median HIT-6 score was 46 (range 36–78). Fifty-one patients (59%) suffered “little or no impact” from their headache; 35 patients (41%) had PTHs, and of these, 25 patients (29%) suffered “severe or substantial impact” as defined by the HIT-6 score (Figure 2A).

Of the 21 patients who sustained moderate/severe TBI, seven (33%) suffered a severe impact from their PTH, whereas of the 55 patients with mild TBI, only 10 (18%) suffered a severe impact of headache (Figure 2A). Consequently, we found that there was a trend to a greater severity of headache disability in those with a more severe head injury.

The median PCL-C score was 26 (range 17–79). In our study, 35 participants (41%) had a PCL-C score that indicated a diagnosis of PTSD. Among those with substantial or severe headache disability (HIT-6 score >56), the majority also had PTSD (72%). Among those with low headache disability (HIT-6 score <49), only 24% had PTSD (Figure 2B).

Thirty participants (35%) had discontinued full-time education after high school, 29 (34%) were employed, 12 (14%) were retired, and 12 were students.

Thirty three patients (38%) suffered a LOC, either at the time of the injury or on admission. The median GCS on presentation was 14 (range 3–15), the median GOS was 5 (range 3–5), and only four patients (<5%) had a seizure. The median Marshall grade of the CTs of these patients was 2 (range 1–6, mode 2). Falls were the most common mechanism of TBI (34 patients; 40%) in this study.

**Figure 3:**
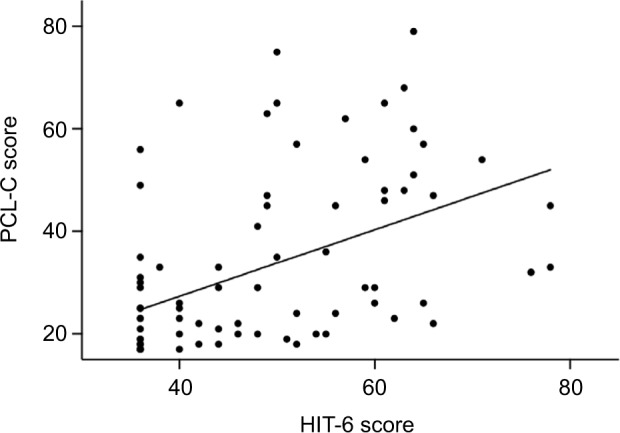
HIT-6 and PCL-C scores correlated significantly (rho=0.583, *p*<0.001). **Abbreviations:** HIT-6, Headache Impact Test version 6; PCL-C, PTSD Check List Civilian version; PTSD, posttraumatic stress disorder.

We assessed factors that were associated with headache disability. PTSD was significantly correlated with headache disability (HIT-6; rho=0.583, *p*<0.001; Figure 3). Moreover, LOC was associated with headache disability (*p*=0.016). Age was negatively correlated with HIT-6 scores (rho=−0.361, *p*=0.001). Employment status was significantly associated with both headache disability and PTSD symptoms (*p*=0.049 and 0.046, respectively). MOI was associated with PTSD symptoms (*p*=0.021), but not headache disability. No significant association between headache disability and the following factors was found: gender, educational level, ethnicity, interval from admission to completion of the questionnaire, seizures, GOS, presence of alcohol consumption, MOI, and Marshall CT grade. After applying a Bonferroni correction, only age and PTSD were significantly correlated with headache disability. Data were further evaluated in a multiple linear regression model, which highlighted that it was predominantly the PCL-C score (*p*<0.001) that explained the variability in the HIT-6 score. Furthermore, age was a secondarily significant variable in the multiple linear regression model (*p*=0.002).

## Discussion

In this study of patients with TBI, we primarily evaluated the relationship between headache disability and PTSD symptoms. In addition, we analyzed patient demographics and variables at the time of injury. Patients in this study were evaluated at a median time of 5.6 months (170 days) post-injury, which is consistent with the diagnosis of chronic PTH (defined as a PTH continuing after 3 months).^1^ As hypothesized, we found that, in our cohort of patients, the factor most significantly associated with headache disability was the presence of comorbid PTSD symptoms. This is in keeping with studies of military patients where the Migraine Disability Assessment Score has been found to be associated with the presence of PTSD, despite these military patients sustaining more severe head injuries than patients in our cohort (64% LOC vs 38% LOC in our cohort).^3^ Our study identifies a comorbidity prevalence rate of 40% for PTSD symptoms and PTH, which concurs with the results reported from another recent work.^9^

Contrary to the findings of Walker et al,^4^ we did not find any association between gender and PTH severity. Despite having a similar gender distribution as our study, the study by Walker et al showed that women reported headache symptoms with 1.89 times more severity than men.^4^ However, Walker et al used a different tool to calculate the “headache density”, whereas we quantified the disability associated with our cohort’s headaches.^17^ Similarly, Ahman et al found that PTSD was more common in women than in men, and that the grading of women’s symptoms was likely to be higher than that of men’s symptoms.^12^ This was not the case in our study cohort as PCL-C scores were evenly distributed across the genders.

Furthermore, our additional analyses highlighted that age impacted on PTH. This is in agreement with the results reported by Lucas et al^6^ who found that patients older than 60 years were significantly less likely to report PTH. The mean age of their cohort and ours is similar (44–46 and 45 years, respectively).^6^ Building on this finding, we show that older age correlated significantly with decreased PTH severity. To ascertain why this is the case, further work is needed; however, we suggest that it may be because primary headaches are also less common in the elderly^26^ or because of lower reporting of headaches in the elderly.

This study is limited by the collection of data at a single time point, and future studies evaluating longitudinal changes will be valuable. Moreover, it is important to understand that although we have identified that the degree of headache disability after brain trauma is associated with PTSD symptoms, this does not imply causation; however, it would be of interest to evaluate whether treatment of PTSD improves headache disability. Furthermore, it would have been interesting to note familial and prior history of migraine, although this was not the primary focus of this study. In future research, a collection of prospective headache diaries, which includes monitoring of headache frequency and severity over time in relation to severity of PTSD symptoms, would be valuable.

## Conclusion

We demonstrate, in a population with predominant mild TBI, that headache disability is greatest in those with PTSD symptoms. This is in agreement with studies suggesting that emotional health can impact headache disability.^27^ Moreover, headache disability is increased in individuals who are younger or lose consciousness at the time of injury. We suggest that in patients with chronic PTH, identifying and treating PTSD may be beneficial for headache management.

### Clinical implications

- Patients with chronic PTH have greater headache disability if they also have PTSD.
- Younger patients who lose consciousness at the time of injury may have the most disability from chronic PTH.
- In patients with chronic PTH and PTSD, headache disability might be improved if PTSD is actively managed alongside headache management.

## Figures and Tables

**Table 1:** Participant demographics

Demographics	n (%)
Gender	
Male	65 (76)
Age*	45 (20)
Education	
High school	30 (35)
University	12 (14)
Unknown	44 (51)
Ethnicity	
White British	65 (76)
South Asian	5 (6)
Black and white mixed Caribbean	1 (1)
African	1 (1)
Oriental Asian	1 (1)
Unknown	13 (15)
Employment status	
Professional	10 (12)
Retired	12 (14)
Semiprofessional	6 (7)
Skilled laborer	13 (15)
Student	12 (14)
Unemployed	11 (13)
Unknown	22 (26)
Interval from admission to completing questionnaireŦ	170 (16–1305)
Loss of consciousness	33 (38)
Seizure	4 (5)
Glasgow Coma ScaleŦ	14 (3–15)
Glasgow Outcome ScaleŦ	5 (3–5)
Alcohol consumption	21 (24)
Mechanism of injury	
Fall	34 (40)
Road traffic accident	23 (27)
Attack	2 (2)
Home accident	20 (23)
Self-harm	1 (1)
Sports injury	3 (4)
Unknown	3 (4)
Marshall CT gradeŦ	2 (1–6)
1	14
2	42
3	6
4	2
5	2
6	12
Unknown	8
Severity of injury♦	
Mild	55 (64)
Moderate/severe	21 (24)
Unknown	10 (12)
HIT-6 scoreŦ	46 (36–78)
Little or no impact	51 (59)
Limited impact	10 (12)
Substantial impact	5 (6)
Severe impact	20 (23)
PCL-C scoreŦ	26 (17–79)
Positive PTSD diagnosis	35 (41)

**Notes:**

* Age: mean and standard deviation are shown.

Ŧ Median and range are shown for interval from admission to completion of questionnaire, Glasgow Coma Scale, Glasgow Outcome Scale, Marshall CT grade, HIT-6 score, and PCL-C score.

♦ As defined by the International Classification of Headache (Beta 3 version).

**Abbreviations:** CT, computed tomography; HIT-6, Headache Impact Test version 6; PCL-C, PTSD Check List Civilian version; PTSD, posttraumatic stress disorder.

## Abbreviations

CT: computed tomography
DSM-IV: *Diagnostic and Statistical Manual of Mental Disorders*, version IV
GCS: Glasgow Coma Scale
GOS: Glasgow Outcome Scale
HIT-6: Headache Impact Test version 6
LOC: loss of consciousness
MOI: mechanism of injury
PCL-C: PTSD Check List Civilian version
PTH: posttraumatic headache
PTSD: posttraumatic stress disorder
TBI: traumatic brain injury
